# Mechanical circulatory support in pediatric patients with biventricular and univentricular hearts

**DOI:** 10.1016/j.xjon.2021.03.002

**Published:** 2021-03-10

**Authors:** Marcus Granegger, Thomas Schlöglhofer, Julia Riebandt, Gerald Schlager, Keso Skhirtladze-Dworschak, Erwin Kitzmüller, Ina Michel-Behnke, Günther Laufer, Daniel Zimpfer

**Affiliations:** a Department of Cardiac Surgery, Pediatric Heart Center Vienna, Medical University of Vienna, Vienna, Austria; b Ludwig–Boltzmann Institute for Cardiovascular Research, Vienna, Austria; c Division of Neonatology, Pediatric Intensive Care, and Neuropediatrics, Medical University of Vienna, Vienna, Austria; d Department of Anaesthesia, Intensive Care Medicine, and Pain Medicine, Medical University of Vienna, Vienna, Austria; e Division of Pediatric Cardiology, Pediatric Heart Center Vienna, Medical University of Vienna, Vienna, Austria

**Keywords:** mechanical circulatory support, single ventricle, pediatric patients, univentricular patients, cf-VAD, continuous-flow ventricular assist device, IQR, interquartile range, MCS, mechanical circulatory support, p-VAD, pulsatile ventricular assist device

## Abstract

**Background:**

Mechanical circulatory support (MCS) in pediatric patients remains challenging because of small body size, limited availability of approved devices, and the variety of etiologies, including biventricular and univentricular physiologies. We report our single-center experience with MCS in pediatric patients in terms of survival and adverse events.

**Methods:**

Outcome, etiologic, and demographic data of pediatric patients implanted with a long-term MCS device between 2011 and 2019 at the Medical University of Vienna were retrospectively collected and analyzed. Overall survival and freedom of treatment-related adverse events at 1 year were investigated by Kaplan–Meier analyses and stratified for circulation (biventricular vs univentricular), age group (<6 years vs >6 years), and pump technology (pulsatile ventricular assist device [p-VAD] vs continuous flow pump [cf-VAD]).

**Results:**

One-year survival of all 33 pediatric patients (median, 4 years; interquartile range, 0-13 years) was 73%, with a tendency toward better outcomes in patients with biventricular circulation than in those with univentricular circulation (80%; n = 25 vs 50%; n = 8; *P* = .063). The trends toward better survival probability in older patients and in patients with cf-VADs did not reach statistical significance (63.2% vs 85.7%; *P* = .165 and 82.4% vs 62.5%; *P* = .179, respectively). Freedom from adverse events was higher in older patients (57.1% vs 5.6%; *P* < .001) and in the cf-VAD group (52.9% vs 0%; *P* < .001), with pump thrombosis as the main discriminator.

**Conclusions:**

MCS is a promising therapy for a broad spectrum of pediatric patients, irrespective of heart failure etiology, age, and pump type. With increasing experience, improved devices, and patient selection, MCS may become a valuable treatment option for patients with univentricular hearts.

## Graphical abstract

**Figure:**
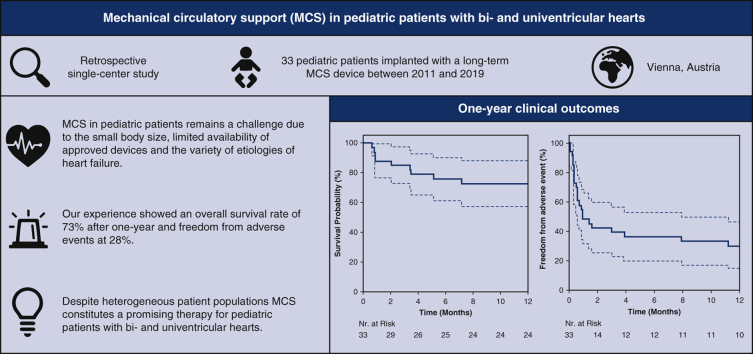

**Figure:**
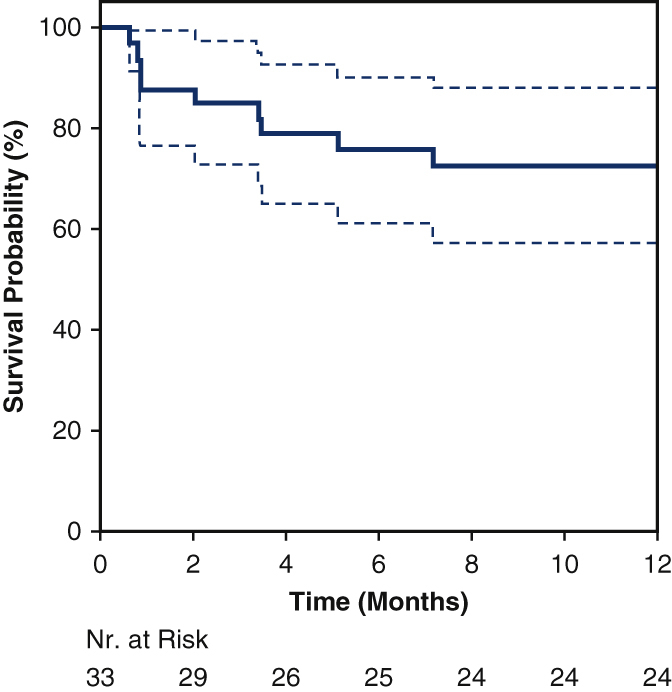
Survival probability of biventricular and univentricular pediatric MCS patients.

**Central Message**

The 1-year survival of 33 pediatric patients with mechanical circulatory support (median age, 4 years; IQR, 0-13 years) was 73%, with a tendency toward better outcomes in biventricular circulation than in univentricular circulation.

**Perspective**

Mechanical circulatory support (MCS) is a promising therapy for a broad spectrum of pediatric patients irrespective of heart failure etiology, age, and pump type. With increasing experience, improved devices, and patient selection, MCS may become a valuable treatment option for patients with univentricular hearts.

See Commentary on page 209.

Medical progress in the past decades has facilitated the survival of children born with univentricular heart diseases until adulthood, resulting in a rising number of patients with univentricular circulation.^1^ However, mortality and failure rate of the univentricular circulation remain of concern; a mortality rate of approximately 30% before the Fontan procedure and a Fontan failure rate of 20% to 30% at 20 years after Fontan completion emphasize the need for further progress in treatment options at each of the palliation stages.^2^^,^^3^

Heart transplantation remains the sole long-term treatment option in these patients. However, the surgical complexity, aspects of immunology after multiple heart surgeries, and long-term impact on liver function, as well as donor availability, contribute to the controversy around outcomes and impede the widespread use of heart transplantation. Compared with patients with biventricular hearts, in patients with congenital heart defects, a significantly longer expected waiting time to transplantation and a higher risk of cardiovascular death on the waiting list^4^ underpin the need for an alternative treatment approach.

Durable mechanical circulatory support (MCS) is considered a promising treatment approach to bridge the patient to transplantation or as destination therapy.^4^, ^5^, ^6^ So far, low patient numbers, a heterogenous patient population, and the variety of intracorporeal and paracorporeal devices in use have made a systematic analysis difficult. Overall reported survival rates for pediatric and adult single-ventricle patients range from 30% to 60%.^7^, ^8^, ^9^ Weinstein and colleagues^10^ showed that the ability to be bridged to transplantation or recovery is lower in univentricular pediatric patients compared with biventricular patients (42.3% [n = 26] vs 72.5% [n = 255]) with the Berlin Heart EXCOR device (Berlin Heart, Berlin, Germany).

Interpretation of results in the pediatric univentricular cohort remains difficult, however. Outcomes in pediatric patients with MCS are affected by age, with better results in older children^11^ and at the palliation stage in univentricular patients with favorable outcomes after Glenn surgery or completion of the Fontan circulation rather than the Norwood/Sano procedure only.^10^ Moreover, the reason for failure of the univentricular circulation, either a failing systemic ventricle or subpulmonary failure^12^ (related to lung performance, ventilation, and vasculature), contributes to a variety of surgical procedures and locations for device implantation.^5^ Additional clinical experience and data are needed before conclusions can be drawn regarding the potential of this approach to successfully bridge patients to transplantation and to identify risk factors to improve patient selection and operative timing.

The aim of this study was to analyze pediatric MCS patients with univentricular or biventricular circulation to explore survival and adverse events with respect to age and device type.

## Methods

Following approval from the local Ethical Committee (EK-Nr. 1769/2018, April 21, 2020) with waiver of informed consent, demographic, etiologic, and clinical outcome data of all pediatric patients (age <18 years) implanted with a long-term MCS device between 2011 and 2019 were retrospectively collected and analyzed. Patients received either a pulsatile assist device (p-VAD) (EXCOR) or a continuous-flow assist device (cf-VAD) (HVAD; Medtronic, Minneapolis, Minn or HeartMate II; Abbott, Indianapolis, Ill).

In consideration of the underlying problem, patients received a ventricular assist device (VAD) supporting the systemic ventricle, the right ventricle, or the systemic and pulmonary circulation (biventricular VAD). All pumps were implanted via a median sternotomy. All left VADs were implanted in an apical configuration, pumping blood from the systemic ventricle to the ascending aorta. For right VADs in patients with biventricular hearts, the right atrium was cannulated to drain venous blood and direct it toward the pulmonary artery. In patients with a univentricular heart, cavopulmonary support was established with custom-made graft adaptations to redirect systemic venous blood with the EXCOR pump from the central veins to the pulmonary arteries as described previously.^13^

The primary endpoint of this study was 1-year survival (including patients undergoing heart transplantation, patients weaned from a device, and patients on a device) for the entire cohort. Treatment failure with respect to the primary endpoint was defined as death; transplantation and permanent pump deactivation for myocardial recovery were not considered treatment failure events. Survival was stratified by circulation (univentricular vs biventricular), device type (p-VAD vs cf-VAD), and age group (0-5 years vs ≥6 years). The threshold of 6 years was chosen based on previous pediatric MCS studies.^14^

As a secondary endpoint, any first treatment-related adverse event was collected for a period of 1 year after implantation. Treatment-related adverse events were defined as pump-related bleeding (gastrointestinal, nonsurgical, and surgical episodes), thromboembolic events (suspected or confirmed pump thrombosis, arterial thromboembolism), and neurologic events including stroke (hemorrhagic or ischemic) and other events (eg, transient ischemic attack, seizure).^15^ If a patient had multiple events, the event that occurred first was noted in the analysis of the secondary endpoint.

Statistical analyses were performed with SPSS for Windows 26.0.0 (IBM, Armonk, NY). Descriptive statistics are presented as mean ± SD for continuous variables and as number (percentage) for categorical variables. Where continuous variables were non-normally distributed, data are presented as median and interquartile range (IQR). Normal distribution was assessed by the Shapiro–Wilk test. Fisher's exact test was used to assess for the statistical significance of categorical variables, and the Student *t* test or Mann–Whitney *U* test was used for continuous variables. Time-to-event analysis was performed using Kaplan–Meier curves, with *P* values reported using the log-rank test. Statistical significance was assumed at *P* < .05.

## Results

### Demographics

Thirty-three pediatric patients (median age, 4 years; IQR, 0-13 years) were included in this retrospective study, including 25 patients with a biventricular physiology and 8 patients with a univentricular physiology. All but 1 patient (who had a HeartMate II) received either the cf-VAD HVAD or p-VAD pump. Despite a trend toward older patients in the cohort with biventricular hearts, the demographic characteristics of the 2 cohorts were comparable (Table 1).

**Table 1:** Demographic and device characteristics of the study population

Variable	All (N = 33)	Biventricular physiology (N = 25)	Univentricular physiology (N = 8)	*P* value
Device, n (%)				.76
Berlin Heart EXCOR	16 (48.5)	11 (44.0)	5 (62.5)	
Medtronic HVAD	16 (48.5)	13 (52.0)	3 (37.5)	
Abbott HeartMate II	1 (3)	1 (4.0)	0 (0)	
Type, n (%)				.65
LVAD	25 (75.8)	18 (72)	7 (87.5)	
RVAD	1 (3)	1 (4)	0 (0)	
BiVAD	7 (21.2)	6 (24)	1 (12.5)	
Age, y, median (IQR)	4 (0-13)	9 (0-13.5)	3.5 (0.75-4.75)	.49
Female sex, n (%)	17 (51.5)	15 (60)	2 (25)	.12
Body mass index, kg/m^2^, median (IQR)	15.35 (13.36-17.55)	15.64 (13.33-18.81)	14.24 (13.38-16.69)	.68
Body surface area, m^2^, median (IQR)	0.66 (0.41-1.35)	1.07 (0.41-1.4)	0.60 (0.41-0.67)	.34
INTERMACS level, n (%)				1
1	22 (66.7)	17 (68)	5 (62.5)	
2	8 (24.2)	5 (20)	3 (37.5)	
3-7	3 (9.1)	3 (12)	0 (0)	

*P* values are provided for the comparisons between the univentricular and biventricular cohorts. *LVAD*, Left ventricular assist device; *RVAD*, right ventricular assist device; *BiVAD*, biventricular ventricular assist device; *IQR*, interquartile range; *INTERMACS*, Interagency Registry for Mechanically Assisted Circulatory Support.

Etiology and palliation stage at implantation of the univentricular cohort are summarized in Table 2. One-half (50%) of univentricular patients had hypoplastic left heart syndrome. Implantation was done after stage 1 (Norwood) palliation in 1 patient, after bidirectional Glenn (stage 2) in 4 patients, and after total cavopulmonary connection (TCPC) in 3 patients with a failing Fontan circulation. Seven univentricular patients received a subaortic VAD, and an EXCOR biventricular VAD (subpulmonary and subaortic support) was implanted in 1 patient with a failing Fontan circulation.

**Table 2:** Etiology and surgical procedure history of patients with univentricular hearts (N = 8)

Procedure history/etiology	Univentricular physiology, n (%)
Procedure history	
Norwood	1 (12.5)
Glenn	4 (50)
Fontan (TCPC)	3 (37.5)
Etiology	
Hypoplastic left heart syndrome	4 (50)
Tricuspid atresia	3 (37.5)
Imbalanced AVSD	1 (12.5)

*TCPC*, Total cavopulmonary connection; *AVSD*, atrioventricular septal defect.

### Overall Survival

**Figure 1:**
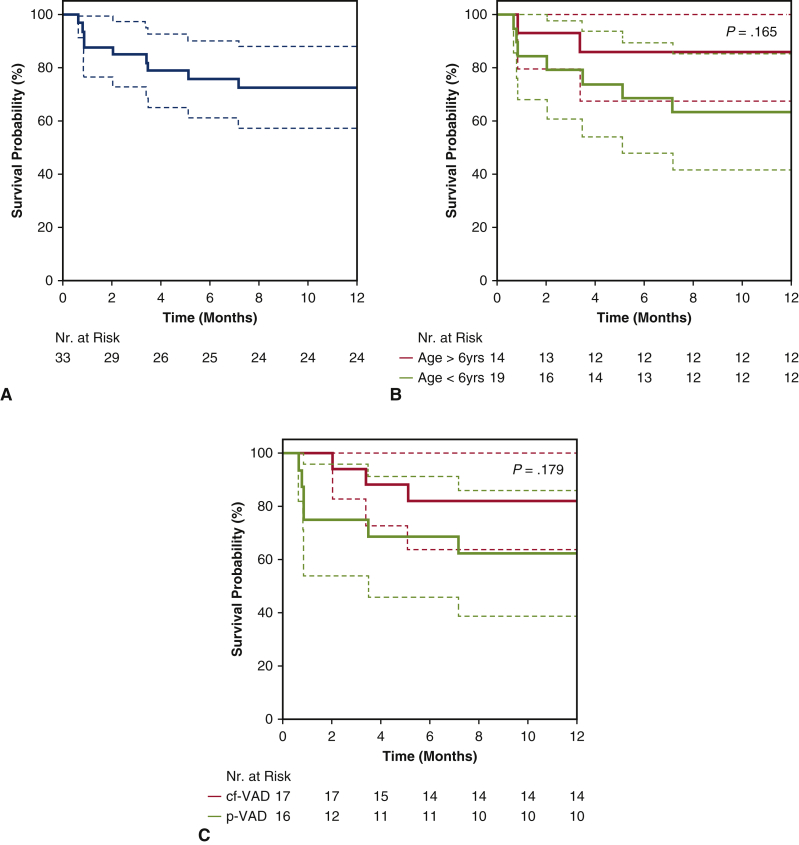
A, Survival probability for all pediatric MCS patients. B, Survival probability stratified by age group. C, Survival probability stratified by pump technology (cf-VAD or p-VAD).

One-year overall survival (OS) in the entire pediatric cohort was 73% (Figure 1, *A*), with a tendency toward better results in the biventricular cohort compared with the univentricular cohort (80% vs 50%; *P* = .063).

#### Influence of age

Despite a trend toward better OS in older patients, no statistically significant difference in OS stratified by age group was observed (63.2% vs 85.7%; *P* = .165) (Figure 1, *B*).

#### Influence of pump type

Concerning the impact of pump type, no statistically significant difference in OS between patients with a cf-VAD and those with a p-VAD were observed in the total cohort (82.4% vs 62.5%; *P* = .179) (Figure 1, *C*). Of note, the patients supported with a p-VAD were significantly younger than those with a cf-VAD (median, 1.7 years [IQR, 0-9 years] vs 11.6 years [IQR, 2-18 years]; *P* < .001), had a smaller BSA (median, 0.45 m^2^ [IQR, 0.23-0.94 m^2^] vs 1.35 [IQR, 0.49-2.93 m^2^], *P* < .001), and were sicker (Interagency Registry for Mechanically Assisted Circulatory Support *I*: 87.5% vs 47.1%; *P* = .037).

### Adverse Events

**Figure 2:**
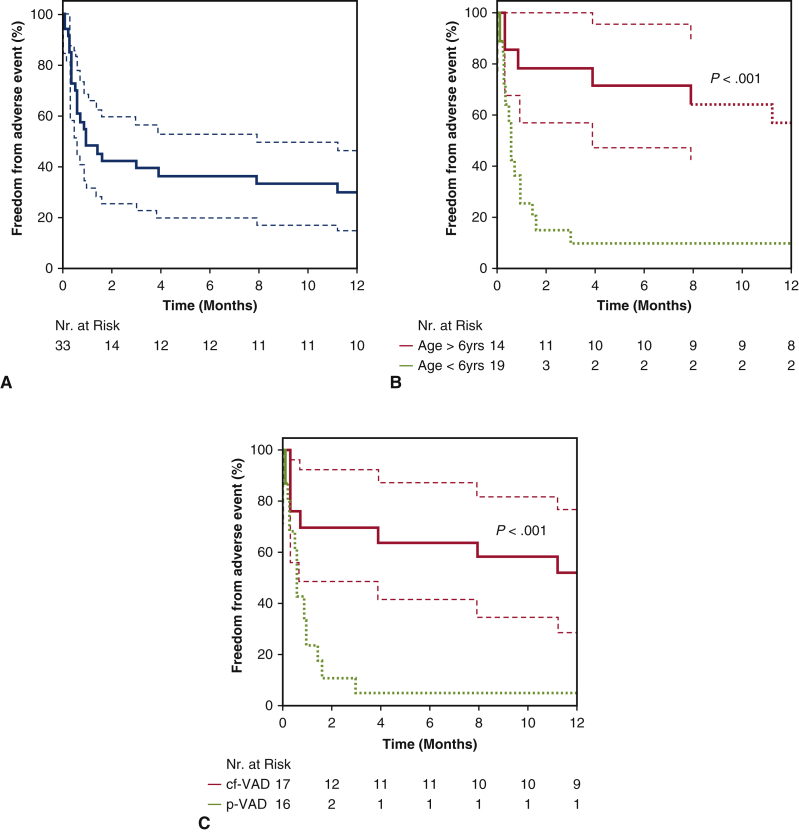
A, Freedom from adverse events for all pediatric MCS patients. B, Freedom from adverse events stratified by age group. C, Freedom from adverse events stratified by cf-VAD or p-VAD. *Dashed lines* indicate fewer than 10 subjects at risk.

More than one-quarter (28.1%) of all patients had no treatment-related adverse events after 1 year (Figure 2, *A*), with no statistically significant difference between the biventricular and univentricular cohorts (28.6% vs 28.0%; *P* = .72). Details regarding the frequency of typical adverse events are summarized in Table 3.

**Table 3:** Freedom from adverse events for the total cohort and stratified by pump type

Freedom from	All (N = 33), %	cf-VAD cohort (N = 17), %	p-VAD cohort (N = 15), %	*P* value
Bleeding	46.9	50	45.7	.817
Ischemic stroke	81.8	87.5	75	.320
Hemorrhagic stroke	87.9	93.8	81.3	.223
Any neurologic dysfunction	72.7	75	68.8	.404
Pump thrombosis	60.6	87.5	31.3	<.05

*P* values are for the comparisons between patients with cf-VADs and those with p-VADs. *cf-VAD*, Continuous-flow ventricular assist device; *p-VAD*, pulsatile ventricular assist device.

#### Influence of age

Older patients had a higher freedom from adverse events (57.1% in patients age ≥6 years vs 5.6% in those age 0-5 years; *P* < .001).

#### Influence of pump type

Freedom from treatment-related adverse events was statistically significantly lower in the p-VAD cohort compared with the cf-VAD cohort (0% vs 53%; *P* < .001), with pump thrombosis the sole difference between the pump technologies (Figure 2, *C* and Table 3).

## Discussion

**Figure 3:**
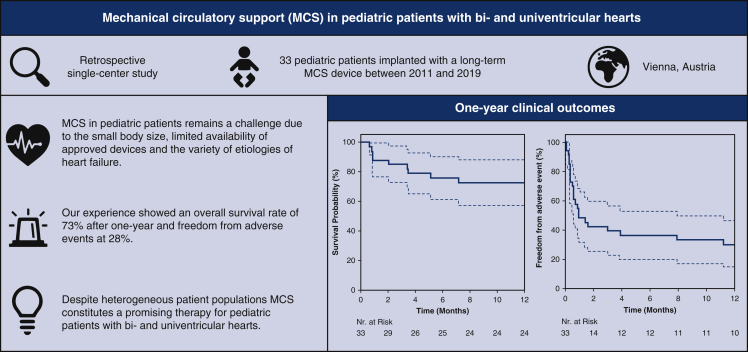
Summary of the study and its most important results.

In this study, 73% of the pediatric patients treated with implantable MCS at our single center achieved a successful outcome in terms of 1-year survival (Figure 3). A trend toward better outcomes was observed in the biventricular cohort (80%, vs 50% in the univentricular cohort). These results are in line with previously published data. Weinstein and colleagues^10^ reported a lower rate of successful outcomes in univentricular patients treated with EXCOR compared with biventricular patients (42.3% vs 72.5%).^10^

No difference was observed between the biventricular and univentricular cohorts in terms of freedom from treatment-related adverse events after 1 year. However, younger patients were at higher risk for adverse events, which may be attributed to the predominant use of p-VADs and the challenging anticoagulation therapy in this population. Patients treated with p-VADs had a statistically significantly higher pump thrombosis rate. All other adverse events were similar in the 2 pump groups. It is noteworthy that the higher pump thrombosis rate in patients with paracorporeal p-VADs did not lead to an increased thromboembolic event rate because of pump exchanges performed immediately after diagnosis of pump thrombosis. However, frequent pump inspection for thrombus formation in p-VADs by experienced VAD clinicians is necessary.

This experience underpins the fact that, although currently available cf-VADs are not designed for the pediatric population,^16^ their benefits in terms of hospital discharge and quality of life remain undeniable. Therefore, in our center we tend to implant cf-VADs after careful evaluation of surgical feasibility in terms of size and implantability.

In general, our findings are in line with previous reports of pediatric MCS patients, indicating that outcomes are affected by device and patient factors, which are highly interdependent. Therefore, much of the difference in outcomes stratified by etiology or VAD technology also may be related to patient characteristics. In addition to preoperative patient status, timing of device placement as well as the underlying disease, age, and weight may be risk factors for death in pediatric MCS patients.^14^^,^^17^

Although this study has some limitations, including its retrospective design, the limitation of data collection to available variables in the medical records, and the analysis of patients from a single center, our experience stresses the greater surgical complexity in patients with univentricular physiology, attributed to the heterogeneity of the patient population, multiple reoperations, and palliation stage. In our experience, MCS has been successfully used in patients at all palliation stages and of all ages (60% for pre–total cavopulmonary connection vs 33% for post–total cavopulmonary connection; *P* = .374). Nevertheless, the heterogeneity of the univentricular population makes it difficult to interpret the results of this single-center experience. Larger studies are needed to provide a more detailed analysis of the effects of such factors as age, palliation stage, and type of MCS. In this study, in all patients with univentricular physiology, the systemic ventricle was supported, and concomitant cavopulmonary support was attempted in 1 patient. Because patients with univentricular hearts often develop dysfunction of the systemic left ventricle and/or subpulmonary failure,^12^ further research is needed. Innovative approaches facilitating subpulmonary support with existing or novel devices tailored to patient's anatomy and physiology are currently in development.^18^, ^19^, ^20^, ^21^

## Conclusions

In heterogenous patient populations with different etiologies, ages, and pump types, MCS represents a promising palliative or bridging therapy for pediatric patients. With increasing experience, improved devices, and better patient selection, MCS may become a valuable treatment option in patients with failing univentricular circulation.

### Conflict of Interest Statement

M.G. has received personal fees and grants from BerlinHeart; T.S. has received personal fees from Medtronic and Abbott; G.L. has received grants, personal fees, and nonfinancial support from 10.13039/100004374Medtronic and 10.13039/100000046Abbott; and D.Z. has received grants, personal fees, and nonfinancial support from 10.13039/100004374Medtronic and 10.13039/100000046Abbott. All other authors reported no conflicts of interest.

The *Journal* policy requires editors and reviewers to disclose conflicts of interest and to decline handling or reviewing manuscripts for which they may have a conflict of interest. The editors and reviewers of this article have no conflicts of interest.

## References

[1] Schilling C., Dalziel K., Nunn R., Du Plessis K., Shi W.Y., Celermajer D. (2016). The Fontan epidemic: population projections from the Australia and New Zealand Fontan Registry. Int J Cardiol.

[2] Shi W.Y., Yong M.S., McGiffin D.C., Jain P., Ruygrok P.N., Marasco S.F. (2016). Heart transplantation in Fontan patients across Australia and New Zealand. Heart.

[3] Ohye R.G., Schonbeck J.V., Eghtesady P., Laussen P.C., Pizarro P., Shrader P. (2012). Cause, timing, and location of death in the single ventricle reconstruction trial. J Thorac Cardiovasc Surg.

[4] Everitt M.D., Donaldson A.E., Stehlik J., Kaza A.K., Budge D., Alharethi R. (2011). Would access to device therapies improve transplant outcomes for adults with congenital heart disease? Analysis of the United Network for Organ Sharing (UNOS). J Heart Lung Transplant.

[5] Miller J.R., Lancaster T.S., Callahan C., Abarbanell A.M., Eghtesady P. (2018). An overview of mechanical circulatory support in single-ventricle patients. Transl Pediatr.

[6] Griselli M., Sinha R., Jang S., Perri G., Adachi I. (2018). Mechanical circulatory support for single ventricle failure. Front Cardiovasc Med.

[7] Arnaoutakis G.J., Blitzer D., Fuller S., Eckhauser A.W., Montenegro L.M., Rossano J.W. (2017). Mechanical circulatory support as bridge to transplantation for the failing single ventricle. Ann Thorac Surg.

[8] Maeda K., Nasirov T., Yarlagadda V., Hollander S.A., Navaratnam M., Rosenthal D.N. (2020). Single ventricular assist device support for the failing bidirectional Glenn patient. Ann Thorac Surg.

[9] VanderPluym C.J., Cedars A., Eghtesady P., Maxwell B.G., Gelow J.M., Burchill L.J. (2018). Outcomes following implantation of mechanical circulatory support in adults with congenital heart disease: an analysis of the interagency registry for mechanically assisted circulatory support (INTERMACS). J Heart Lung Transplant.

[10] Weinstein S., Bello R., Pizarro C., Fynn-Thompson F., Kirklin J., Guleserian K. (2014). The use of the Berlin Heart EXCOR in patients with functional single ventricle. J Thorac Cardiovasc Surg.

[11] VanderPluym C.J., Adachi I., Niebler R., Griffiths E., Fynn-Thompson F., Chen S. (2019). Outcomes of children supported with an intracorporeal continuous-flow left ventricular assist system. J Heart Lung Transplant.

[12] Murtuza B., Hermuzi A., Crossland D.S., Parry G., Lord S., Hudson M. (2017). Impact of mode of failure and end-organ dysfunction on the survival of adult Fontan patients undergoing cardiac transplantation. Eur J Cardiothorac Surg.

[13] Prêtre R., Häussler A., Bettex D., Genoni M. (2008). Right-sided univentricular cardiac assistance in a failing Fontan circulation. Ann Thorac Surg.

[14] Morales D.L.S., Adachi I., Peng D.M., Sinha P., Lorts A., Fields K. (2020). Fourth annual pediatric interagency registry for mechanical circulatory support (Pedimacs) report. Ann Thorac Surg.

[15] Uriel N., Colombo P.C., Cleveland J.C., Long J.W., Salerno C., Goldstein D.J. (2017). Hemocompatibility-related outcomes in the MOMENTUM 3 trial at 6 months: a randomized controlled study of a fully magnetically levitated pump in advanced heart failure. Circulation.

[16] Granegger M., Thamsen B., Schlöglhofer T., Lach S., Escher A., Haas T. (2020). Blood trauma potential of the HeartWare ventricular assist device in pediatric patients. J Thorac Cardiovasc Surg.

[17] Morales D.L.S., Rossano J.W., VanderPluym C., Lorts A., Cantor R., St Louis J.D. (2019). Third annual pediatric interagency registry for mechanical circulatory support (Pedimacs) report: preimplant characteristics and outcomes. Ann Thorac Surg.

[18] Granegger M., Thamsen B., Hubmann E.J., Choi Y., Beck D., Valsangiacomo Buechel E. (2019). A long-term mechanical cavopulmonary support device for patients with Fontan circulation. Med Eng Phys.

[19] Granegger M., Schweiger M., Schmid Daners M., Meboldt M., Hübler M. (2018). Cavopulmonary mechanical circulatory support in Fontan patients and the need for physiologic control: a computational study with a closed-loop exercise model. Int J Artif Organs.

[20] Rodefeld M.D., Marsden A., Figliola R., Jonas T., Neary M., Giridharan G.A. (2019). Cavopulmonary assist: long-term reversal of the Fontan paradox. J Thorac Cardiovasc Surg.

[21] Cysyk J., Clark J.B., Newswanger R., Jhun C.S., Izer J., Finicle H. (2019). Chronic in vivo test of a right heart replacement blood pump for failed Fontan circulation. ASAIO J.

